# A Long Temporal Study of Parasitism in Asexual-Sexual Populations of* Carassius gibelio*: Does the Parasite Infection Support Coevolutionary Red Queen Dynamics?

**DOI:** 10.1155/2018/6983740

**Published:** 2018-03-11

**Authors:** Tomáš Pakosta, Lukáš Vetešník, Andrea Šimková

**Affiliations:** ^1^Department of Botany and Zoology, Faculty of Science, Masaryk University, Kotlářská 2, 611 37 Brno, Czech Republic; ^2^Institute of Vertebrate Biology, Academy of Sciences of the Czech Republic, v.v.i., Květná 8, 603 65 Brno, Czech Republic

## Abstract

*Carassius gibelio* is an extraordinary cyprinid species exhibiting both sexual and asexual reproduction. We hypothesized that parasitism selection is one of the potential mechanisms contributing to the coexistence of the two reproductive forms of* C. gibelio* living in the same habitat. We performed a four-year study to investigate the dynamics of parasite infection in* C. gibelio*. According to the Red Queen prediction, the asexual form is a target of parasite adaptation due to its low genetic variability. Both sexual and gynogenetic forms of* C. gibelio* exhibited similar levels of prevalence, with monogeneans being the most frequently observed parasite group. We observed the temporal dynamics of parasite infection in the last year of investigation, when both forms were more strongly parasitized. The sexual form was more parasitized by ectoparasites in the first and last years and less parasitized by nematodes in the last year when compared to the gynogenetic form. We found no trend of high parasite infection in gynogenetic mtDNA haplotypes. We conclude that Red Queen dynamics is not the mechanism driving parasite infection in sexual-gynogenetic* C. gibelio* over a long time scale. Alternatively, we suggest that the dynamics of parasite infection in this complex may be generated by multiple mechanisms.

## 1. Introduction

The coexistence of sexual and asexual forms is rarely reported in vertebrates. The stable coexistence of these two forms may be achieved if there are some disadvantages for asexuals compensating the evolutionary twofold costs of sexual reproduction [1, 2]. The coexistence of asexual and sexual reproduction is documented only in a small number of freshwater fishes including nine genera [3]. In the context of asexual reproduction, gynogenesis has a specific constraint as the sperm of males are necessary to induce embryogenesis; that is, the eggs of gynogenetic females are stimulated by sperm of usually conspecific (or, alternatively, phylogenetically closely related) males to start embryogenesis but the sperm do not fuse with the egg nucleus. Thus, in such case, the asexual and sexual forms are forced to coexist as the persistence of the sexual form is in the direct interest of the gynogenetic form. This means that gynogenetic form (which may be considered as a “parasite” using the sperm of sexual males) cannot outcompete the sexual form (which may be considered as “host” for gynogenetic form).

The* Carassius auratus* complex is one of the rare complexes exhibiting both gynogenetic and sexual reproduction. This nonnative species complex colonized Europe in the second half of the 20th century and is considered to be the most successful invasive species in European waters [4–9], very effectively expanding and adapting to new habitats due to its high ecological tolerance [10]. Representatives of the* C. auratus* complex entered the Czech hydrological system by means of migration from the Danube in around 1975 [7, 11, 12], and over the next 20 years the complex spread to the Danube tributaries, that is, the Morava and Dyje Rivers, where it produced stable populations [13]. The first populations of the* C. auratus* complex were triploid gynogenetic females [14, 15]. Fifteen years after the initial invasion, however, mixed diploid-polyploid populations, including sexual diploid males and females, gynogenetic triploid females, and rarely triploid males or even tetraploid specimens, were reported [16–18]. In recent times, both reproductive forms have coexisted successfully in the same habitats. The particular success of colonization by former unisexual gibel carp populations was the result mainly of gibel carp's specific biological properties, that is, the ability to reproduce gynogenetically, resulting in high reproductive output, but was also due to its own invasive activities, favourable environmental factors, and human activity [14]. It seems that the shift from gynogenesis to sexual reproduction followed the stabilization of* C. gibelio* populations in the successfully colonized habitats. In the area of the Czech Republic, the* C. auratus* complex is represented by four mitochondrial lineages:* C. gibelio*,* C. auratus*,* C. langsdorfii*, and the so-called M line.* Carassius gibelio* is the most commonly occurring form of this complex in the Czech Republic [19].

Several mechanisms have been studied and suggested as contributing to the coexistence of sexual and asexual forms in fish complexes. Some of them have also been hypothesized and examined with respect to the* C. auratus* complex. The role of parasites in maintaining the coexistence of asexual and sexual reproduction is based on the Red Queen (RQ) hypothesis, which predicts antagonistic coevolutionary host-parasite interactions leading to sustained oscillations in their genotype frequencies [20, 21]. Following the RQ hypothesis, the asexual form is, due to its low genetic recombination, a target of parasite adaptation [22], as parasites adapt quickly to common genotypes [23]. In contrast, the sexual form quickly and effectively produces combinations of parasite-resistant genes and therefore is able to escape infection [22, 24]. In accordance with the RQ hypothesis, Hakoyama et al. [25] showed that parasitism plays an important role in maintaining the coexistence of sexual and gynogenetic forms of the* C. auratus *complex. They reported higher prevalence of the trematode species* Metagonimus* sp. in gynogenetic females when compared to diploid females in natural habitats. In accordance with this observation, Hakoyama and Iwasa [26] concluded that the coexistence of sexual and gynogenetic forms of the* C. auratus* complex is stabilized by parasites. However, the mechanisms predicted by the RQ hypothesis did not explain the coexistence of sexual and asexual forms in other freshwater fish complex, that is,* Cobitis taenia* hybrid complex [27]. In that case, differential habitat preferences proposed by niche shift hypothesis contributed to the coexistence of both forms. In addition, Hakoyama and Iwasa [26] also proposed that the coexistence of* C. auratus* complex is not strictly maintained by parasites but may result from interactions between several factors (i.e., assortative mating, female-biased sex allocation, different dispersion capacities, and different utilization of niches). Šimková et al. [28] examined the parasite load in gynogenetic and sexual* C. gibelio* in the light of the RQ hypothesis. They applied the genotyping of major histocompatibility complex (MHC) IIB genes and showed that the most common MHC genotype of the gynogenetic form is more parasitized than sexual genotypes or rare gynogenetic genotypes. In addition, Šimková et al. [29] hypothesized that the different investments in condition-, growth-, and fitness-related traits may represent other mechanisms contributing to the coexistence of gynogenetic and sexual forms of the* C. auratus* complex. However, they revealed similar growth, expressed in terms of body size, and similar condition factors in gynogenetic and sexual forms and failed to identify the reproductive disadvantage for the gynogenetic form when investment in reproduction was measured by gonad weight and estradiol level. This indicates that similar energy is invested in the reproduction of gynogenetic and sexual females. In addition, Šimková et al. [29] proposed the existence of a new mechanism potentially contributing to the coexistence of gynogenetic and sexual forms of the* C. auratus* complex, that is, the low aerobic performance in gynogens, which may represent a physiological disadvantage balancing the evolutionary cost of sexual reproduction. In addition, Vetešník et al. [30] identified higher concentrations of triacylglycerols and cholesterol in the blood plasma of gynogenetic females, indicating a higher metabolic rate and higher energy intake when compared to sexuals.

Finally, the role of immunity in maintaining the coexistence of sexual and gynogenetic forms of the* C. auratus* complex was examined. While Hakoyama et al. [25] showed lower nonspecific immunity and higher parasite loads of nonspecific trematode parasite species (*Metagonimus* sp.) in the gynogenetic form of the* C. auratus *complex (their study was performed using gynogenetic* C. langsdorfii* and an unspecified sexual line of* C. auratus* complex), Šimková et al. [28, 29] showed no difference in nonspecific immunity between the gynogenetic and sexual forms of* C. gibelio*, but higher specific immunity (IgM level) as well as higher parasite loads of host specific monogeneans (*Dactylogyrus* spp.) in the gynogenetic form when compared to the sexual form.

As the pattern of parasite infection in fish populations composed of asexual and sexual forms had not yet been studied over a long time scale, we focused this study on the temporal dynamics of parasite infection. The aim was to perform a long-term study (over four consecutive years) to investigate the composition of parasite communities and parasite load in asexual and sexual forms of* C. gibelio*.

## 2. Materials and Methods

### 2.1. Fish Sampling

A total of 203 individuals of the* Carassius auratus *complex (standard length 28.35 ± 2.61 cm) were caught by electrofishing in the River Dyje near the city of Břeclav (48°80′N; 16°84′E; the Morava River basin). Fish were sampled during the same period in each of four consecutive years (first two weeks in August, 2012–2015). After blood and fin sampling, fish were immediately placed in a tank containing original water and transported to the laboratory. For each specimen, the total and standard lengths, body weight, and sex were recorded. The complete parasitological dissection of fish was performed following Ergens and Lom [31]. A fin clip of each specimen was preserved in 90% ethanol for molecular analyses.

### 2.2. Parasite Collection and Identification

Monogeneans were removed from the gills or fins, placed into a drop of water on a slide, covered with a coverslip, and fixed using glycerine ammonium picrate (GAP) for later analysis [32]. The remaining parasite species were fixed in 70% ethanol. Parasite specimens were examined using an Olympus model BX50 light microscope (including an Olympus DP71 digital microscope camera) with phase contrast, differential interference contrast, and digital imaging (MicroImage 4.0 for Windows).

### 2.3. Ploidy Determination and Molecular Analyses

A fin clip of about 1 cm^2^ in area was taken from each fish for ploidy detection and fixed in 70% ethanol. Before analysis, this tissue was homogenised on a Petri dish in a 2 ml solution of CyStain DNA 1 step Partec, and relative DNA content was estimated using a Partec CCA I continuous flow cytometer (Partec GmbH; https://www.sysmex-partec.com) as was applied previously for ploidy measurements using blood sample [33, 34]. Fresh blood of diploid* C. auratus* was obtained by puncture of the caudal blood vessel using a heparinised syringe and was used as a reference standard. Overall, for our study, 112 diploid specimens (comprising 56 females and 56 males) and 91 triploid females were randomly selected. The control region of mitochondrial DNA (D-loop) was analysed for each specimen. DNA was extracted by Dneasy™ Blood and Tissue Kit (Qiagen) from fin clips preserved in ethanol. PCR and DNA sequencing were performed according to Sambrook et al. [35] with small modifications following Vetešník et al. [36] and Papoušek et al. [37]. For amplification, the forward primer CarU32 (5′-CCAAAGCCAGAATTCTAAAC-3′) and the reverse primer CarL509 (5′-CATGTGGGGTAATGA-3′) [37] were used. A PCR product of 475–493 bp in length covers all ETAS domains (representing a high degree of variability considered useful for the study of intra- and interspecies evolution) [38] and a part of the central domain in the control region. PCR was performed in a final volume of 30 *μ*l containing 1x PCR reaction buffer, 1.5 mM MgCl_2_, 0.3 *μ*M of each primer (CarU32 and CarL509), 0.2 mM dNTPs, 1.5 U Taq polymerase, and 100 ng/*μ*l of template gDNA. PCR was carried out using the following steps: 1 min of denaturation at 95°C, followed by 32 cycles of 45 s at 94°C, 30 s at 50°C, and 45 s at 72°C, and 10 min of final elongation at 72°C. The PCR products were checked on 1.5% agarose gel, purified by High Pure PCR product purification kit (Roche), and directly sequenced using BigDye Terminator Cycle sequencing kit on an ABI 3130 Genetic Analyser (Applied Biosystems, USA). The sequences were aligned using MEGA 6.0 [39] and analysed using TCS 1.21 [40]. The obtained sequences were compared with the sequences for the mtDNA haplotypes of the* C. auratus *complex available in GenBank. The sequences of mtDNA haplotypes obtained in this study were identical with the sequences available in GenBank under the following accession numbers: FJ167410, FJ167411, FJ167413, FJ167414, FJ167416, FJ167420, and FJ167423–FJ167425.

### 2.4. Statistical Analyses

General linear models (GLM) were used to evaluate the three main effects, that is, ploidy, sex, and year, on parasite abundance, as well as two interaction effects, that is, ploidy with year and ploidy with body size. The standard body length of fish was included as a covariate. Parasite abundance was expressed by the following variables: total parasite abundance (i.e., the abundance including all parasite specimens), total monogenean abundance (i.e., the abundance of the most common parasitic group), total abundance of Nematoda (i.e., the abundance of the most common endoparasite group), and the abundance of the most common parasitic species or genera, that is,* Dactylogyrus *spp. (gill Monogenea),* Gyrodactylus *spp. (gill and fin Monogenea), and* Ichthyophthirius multifiliis* (Protozoa). For multiple comparisons, the Tukey post hoc test was used. All variables applied in this study were examined for normal distribution and homogeneity of variance. For data that did not fit these assumptions log-transformation was applied. Statistical analyses were performed in Statistica for Windows 12, StatSoft Inc.

## 3. Results

Random sampling of the mixed population of* C. gibelio* in the studied locality over a period of four years revealed the following composition of the mixed gynogenetic-sexual population: 38% of fish were triploid specimens (37% were gynogenetic females and 1% were triploid males) and 63% of fish were sexual diploid specimens. Molecular analysis of mtDNA confirmed that all specimens were representatives of the* C. auratus* complex. Our study revealed the presence of 9 variants of 16 mtDNA haplotypes previously described in the Southern Moravian region of the Czech Republic by Papoušek [19] (Table 1). The* Carassius gibelio* lineage was the most frequent in the studied locality (96%). Six mtDNA haplotype variants of* C. gibelio* were identified: G01, G02, G04, G05, G07, and G11, with G02 representing the most common haplotype (Table 1). Two of the other haplotypes, G01 and G04, were also common. For sexual specimens, G02 and G04 were the most common haplotypes and G05 was a rare mitochondrial genotype in all four years of investigation. For gynogenetic females, differences in haplotype frequencies among the four years were observed. The most frequent haplotypes of gynogenetic females reported in each year were as follows: G01, G02, and G07 in 2012; G02 in 2013; and G01 in both 2014 and 2015. Among the 6* C. gibelio* mtDNA haplotypes, 3 haplotypes were found exclusively in gynogenetic females, one was found exclusively in sexual specimens, and two were shared by sexual and gynogenetic specimens. The other three lineages,* C. auratus *(haplotype A01),* C. langsdorfii* (haplotype L01), and M line (haplotype M01), were rare (2.4%, 0.5%, and 1%, resp.). For the following analyses, only the* C. gibelio* lineage was selected due to the high frequency of this form.

Parasite communities consisted of the representatives of 8 taxonomic groups (Table 2). A total of 11 species of Monogenea, 1 species of Cestoda, 3 species of Trematoda, 2 species of Nematoda, 1 species of Hirudinea, 1 species of Mollusca (larval stages termed glochidium), 1 species of Protozoa, and 2 species of Crustacea were identified (Table 2). The parasite infection level (expressed by prevalence, abundance, and intensity of infection) was compared between gynogenetic and sexual forms. The prevalence of* Dactylogyrus* spp. and* Gyrodactylus sprostonae* (Monogenea) was high in sexual males and females as well as in gynogenetic females (maximum prevalence was reported for 5* Dactylogyrus* species and a prevalence of more than 80% for* Gyrodactylus*). More specifically,* Dactylogyrus dulkeiti*,* D. intermedius*,* D. anchoratus*,* D. formosus*, and* D. inexpectatus* and* Gyrodactylus sprostonae* represented the ectoparasite species reaching the highest intensities of infection in both forms. The prevalence of 5 other ectoparasite species, that is,* D. vastator*,* D. baueri*,* G. shulmani*,* G. longoacuminatus*, and* Ichthyophthirius multifiliis*, was moderate. However, the intensity of infection of* I. multifiliis *was also high. The prevalence of other parasite species was low (≤45%). Concerning endoparasite species,* Schulmanela petruschewskii* and* Philometroides sanguinea* (Nematoda) achieved the highest intensity of infection, especially in females (both gynogenetic and sexual). Total parasite abundance as well as monogenean parasite abundance was lower in gynogenetic G07 haplotypes when compared to other haplotypes (Mann–Whitney test, *p* = 0.018 and *p* = 0.008, resp.).

**Figure 1:**
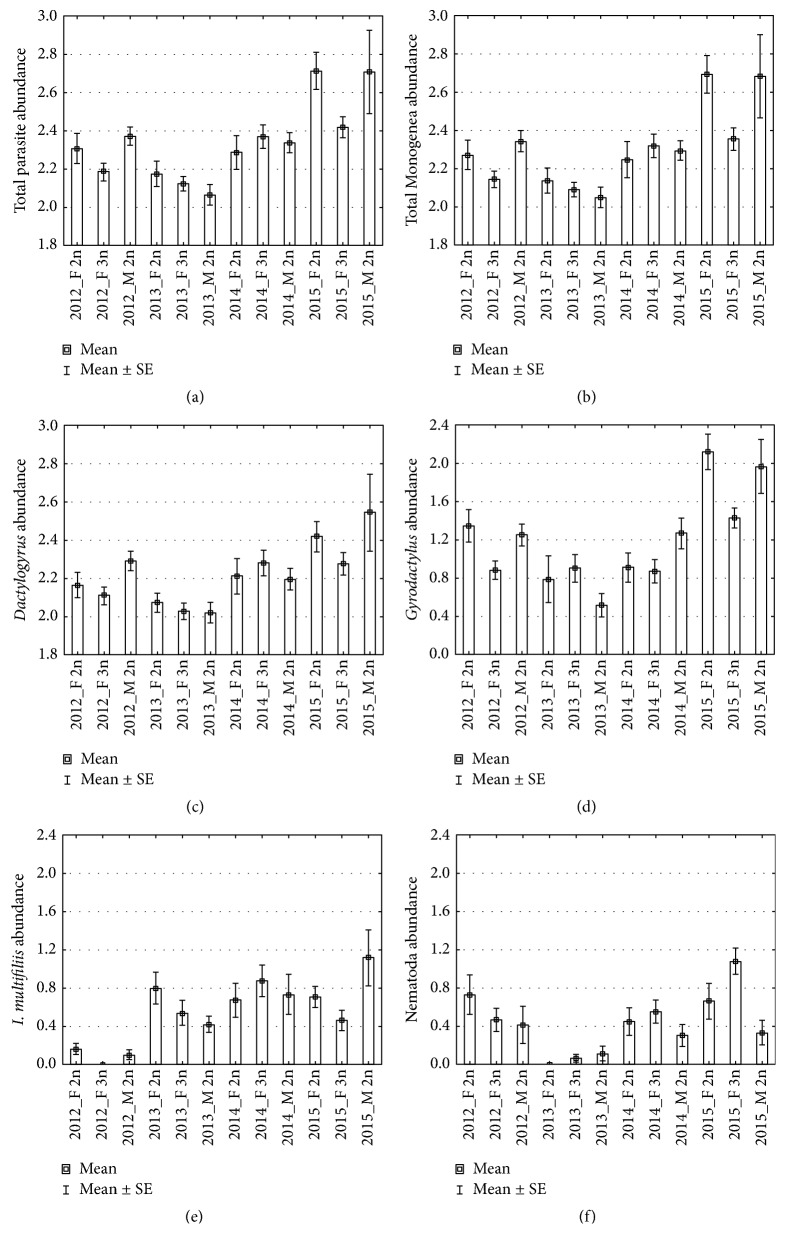
Parasite abundance (transformed in log) in sexual diploid form (F2n: diploid females and M2n: diploid males) and gynogenetic triploid females (F3n). Parasite abundance is expressed using the total parasites (a), Monogenea (b),* Dactylogyrus* species (c),* Gyrodactylus* species (d),* Ichthyophthirius multifiliis* (e), and Nematoda (f).

The effects of ploidy, sex, and year on parasite abundance were analysed (Table 3) as described in Materials and Methods. Total parasite abundance (Figure 1(a)) as well as monogenean abundance (Figure 1(b)) was significantly affected by year, body size, and ploidy (through the interaction effect of ploidy and year). The highest levels of total parasite abundance and monogenean abundance were reported in 2015, the lowest in 2013. Sexual diploids were more parasitized than gynogenetic females in 2012 (*p* < 0.05) and 2015 (*p* < 0.01). However, no significant differences in total parasite abundance or monogenean abundance were found between 2013 and 2014.

*Dactylogyrus* abundance was significantly affected by year and body size and there was also a weak but insignificant effect of ploidy (*p* = 0.127) through the interaction effect of ploidy and year (Figure 1(c), Table 3).* Dactylogyrus* abundance was significantly higher in 2015 compared to the first two years of investigation and was also higher in 2014 compared to 2013. In 2015,* Dactylogyrus* abundance was slightly higher in sexual diploids than in gynogenetic females (*p* = 0.046).* Gyrodactylus* abundance (Figure 1(d)) was significantly affected by year and ploidy through the interaction effect of ploidy and year.* Gyrodactylus* abundance reached its highest values in 2015 and was also higher in 2012 compared to 2013. In 2012 and 2015, sexual diploids were more parasitized by* Gyrodactylus* spp. when compared to gynogenetic females (*p* < 0.01).

The abundance of* I. multifiliis* was significantly affected by year and body size, and there was a small but insignificant effect of ploidy (*p* = 0.14) through the interaction effect of ploidy and year (Table 3, Figure 1(e)). The lowest abundance of* I. multifiliis *was found in 2012. In 2012 and 2015, sexual diploids were more parasitized by* I. multifiliis *when compared to gynogenetic females.

The abundance of Nematoda (two nematode species were pooled for the analysis) was significantly affected by year, ploidy, and body size (Table 3, Figure 1(f)). A significant interaction effect of ploidy and body size on Nematoda abundance was also found. The highest Nematoda abundance was found in 2015 and the lowest in 2013. Overall, females were more parasitized than males (*p* = 0.002) and gynogenetic females were more parasitized than sexual diploids (*p* = 0.010 as evidenced in 2014 and 2015; see Figure 1(f)). However, when the effect of ploidy was analysed within each year, a significant difference in the abundance of Nematoda between gynogenetic females and sexual diploids was found only in 2015 (*p* = 0.004).

## 4. Discussion

The Red Queen hypothesis describes host-parasite dynamics on the basis of negative parasite mediated frequency dependent selection, which can represent one of the advantages of sexual reproduction over asexual reproduction [2, 22, 41, 42]. Thus, we may expect that the asexual form of a species, which exhibits low genetic variability, will suffer from higher parasite load when compared to the sexual form, which exhibits high genetic variability due to recombination. However, as already mentioned, there is no clear empirical support for this hypothesis, as studies focussing on parasite load in coexisting asexual and sexual complexes do not show a consistent pattern. In the case of the gynogenetic-sexual complex including sexual* Poeciliopsis monacha* and the two coexisting gynogenetic triploid clones of* P. 2monacha-lucida*, Lively et al. [22] reported a higher accumulation of the metacercaria stage of the trematode* Uvulifer* sp. causing black spot disease in the most common triploid clone when compared to sexual species and the less common gynogenetic clone on different temporal and spatial scales (i.e., two different years of collection and three different pools). Infection by the metacercaria stage of the trematode* Metagonimus *sp., also forming a black spot on the fish epidermis, was analysed in the gynogenetic-sexual complex of* Carassius auratus langsdorfii*—*C. a. bürgeri *by Hakoyama et al. [25]. In this complex, the gynogenetic form is infrequent. They suggested that the higher parasite load in the gynogenetic form was, in part, due to the lower nonspecific immune reaction in the gynogenetic form compared to the sexual form. In contrast, no consistent pattern in parasite load between asexual* Poecilia formosa* and sexual* P. latipinna* was observed on the spatial scale [43]. In their study, four populations were studied—two exhibiting no difference in parasite load between asexual and sexual species, one population exhibiting high overall parasite load among asexual species, and the last exhibiting a greater micro-parasite load among sexual species.

In our study, we revealed the same composition of parasite communities in both sexual and gynogenetic forms of* C. gibelio*, that is, a high number of parasite species with Monogenea (ectoparasite group exhibiting generally high degree of host specificity) predominant and similar levels of prevalence. Surprisingly, some monogenean species exhibited a trend of higher maximum intensity of infection in sexual specimens when compared to gynogenetic females. This trend was most evident for* Gyrodactylus sprostonae*, representing monogeneans with a viviparous strategy (and very fast reproduction rate) and lower degree of host specificity when compared to oviparous and highly host specific gill monogeneans of* Dactylogyrus*. On the other hand, gynogenetic females tended to be more parasitized with respect to the total number of nematodes (but see below). However, within-year comparisons revealed higher monogenean abundance (for both* Dactylogyrus* and* Gyrodactylus* species) in the sexual form when compared to the gynogenetic form partially for 2012 and particularly for 2015, when the overall level of parasite infection was the highest. No obvious differences in monogenean abundance were found in 2013, and, in 2014,* Dactylogyrus* abundance among gynogenetic females even tended to be slightly higher than* Dactylogyrus *abundance among the sexual form (though the difference was not significant). Concerning endoparasites, Nematoda were the most abundant parasite group, with two species reported in this study—*Philometroides sanguinea* (parasite species restricted to* Carassius* species) and* Schulmanela petruschewskii* (parasitizing a wide range of fish species). The gynogenetic form was more parasitized by nematodes than the sexual form. However, it seems that this result was generated by the effect of body size, as gynogenetic females reached greater body sizes than sexual specimens. During all four years of investigation, gynogenetic females represented 30–40% of the mixed population; sexual females, 35–40%; and sexual males, 22–30%. As a result, the sexual form was more frequent in the habitat than the asexual form, which may partially explain the observed pattern of parasite infection. The coexisting gynogenetic-sexual* C. gibelio* complex was previously investigated by Šimková et al. [28]. They found that there was a small difference in parasite load between sexual and gynogenetic forms (gynogenetic females suffered from slightly higher parasite infection). However, in their study, the selected mixed diploid-triploid population of* C. gibelio* was collected at two-time points (two consecutive years), with coexisting gynogenetic females representing 51–54% of the population, sexual females representing 20–24%, and sexual males representing 20–22%. A different ratio of sexual and asexual forms of* C. gibelio* in a given population, a different population density, or the influence of unexamined abiotic or biotic factors may potentially explain the difference between the results observed in the present study and those obtained previously by Šimková et al. [28], as in the latter study the sampling of* C. gibelio* was performed in a different locality. In addition, in the study by Šimková et al. [28],* Gyrodactylus* parasites represented only a small fraction of the observed parasite communities, while* Dactylogyrus* parasites (with an oviparous strategy, a slower reproduction rate, and higher degree of host specificity when compared to* Gyrodactylus*) predominated. It is also possible that, in some localities, the coexistence of gynogenetic and sexual forms of* C. gibelio* is not solely maintained by parasites and that some other mechanisms may be involved or even act more strongly than parasites. It is also possible that phylogenetically closely related cyprinid species (i.e.,* Carassius carassius* and* Cyprinus carpio*) living in sympatry with* C. gibelio* act as suitable host species for the transmission of host specific parasites in some localities. However, their occurrence was extremely rare in our system studied. The general validity of the RQ hypothesis with respect to how the coexistence of gynogenetic and sexual forms of* C. gibelio* is maintained needs to be verified by examining a wider range of mixed populations with different ratios of gynogenetic and sexual forms likely reflecting the specific stages of evolution of this complex.

Šimková et al. [28] also performed genotyping using the major histocompatibility complex genes (MHC IIB) of gynogenetic and sexual specimens. They hypothesized and confirmed that the most common gynogenetic clone is the target of parasite selection; that is, they showed that the most frequent MHC clones of gynogenetic* C. gibelio* are more parasitized than sexual specimens or gynogenetic specimens with rare MHC genotypes. In the present study, no data concerning MHC genotype diversity was available. Here, we applied an analysis of mitochondrial haplotype diversity (based on D-loop sequence analysis). Concerning the mtDNA haplotypes in* C. gibelio* in the Czech Republic previously studied by Papoušek [19], G02 was the most common. In the locality sampled for this study, G02 was also the most common haplotype reported in the total sample of* C. gibelio* and was more frequently reported in sexual diploids than in gynogenetic triploid females. In accordance with Papoušek [19], we identified G05 haplotype only in sexual diploids and G11 only in gynogenetic females. However, our data revealed G01 as the most frequent haplotype in gynogenetic females (47% of specimens). In contrast to Papoušek [19], we failed to identify this haplotype in sexual diploid form. Concerning overall haplotype diversity, the gynogenetic form exhibited a higher number of haplotypes when compared to the sexual form. When comparing haplotype frequencies among the four years, similar frequencies of haplotypes were identified for the sexual form, while some changes in haplotype frequency were observed for gynogenetic females. However, these changes were not commensurate with changes in parasite infection among years. Parasite abundance was similar in all gynogenetic, sexual, and mixed haplotypes, except for one rare gynogenetic haplotype, namely, G07, which was less parasitized than other haplotypes. This less parasitized haplotype was present mainly in fish sampled in 2012.

## 5. Conclusions

In the present long-term study, we investigated the compositions of parasite communities and levels of parasite infection in coexisting gynogenetic and sexual forms of the cyprinid* C. gibelio*, representing the most common form of the* C. auratus *complex in the Czech Republic. Gynogenetic and sexual forms shared almost all parasite species and, overall, exhibited similar levels of parasite infection. However, the level of parasite infection was variable for both forms over the four years of investigation, potentially as a result of some temporal variability of multiple abiotic and/or biotic factors. We found higher mtDNA haplotype diversity in the gynogenetic form when compared to the sexual form. Our results indicate that changes in the frequencies of mitochondrial genotypes across years do not explain the observed temporal variability in parasite infection. Different patterns of parasite infection in sexual and gynogenetic forms of* C. gibelio* were reported when comparisons were made within individual years, a finding which may potentially be associated with the dynamics of host-parasite interactions. However, the observed dynamics of parasite infection does not support the RQ hypothesis, as we failed to find a significantly higher level of parasite infection in the asexual form in any single year of investigation. With respect to future studies focused on the dynamics of host-parasite interactions, we suggest the need to analyse other genetic markers, especially those related to host immunity.

## Figures and Tables

**Table 1:** Haplotypes of the mitochondrial control region (D-loop) in investigated specimens of *Carassius auratus* complex. Total numbers of specimens and the numbers of specimens in each of four consecutive years (in parentheses) are shown for each mtDNA haplotype.

*C. auratus* complex	Haplotype	Number of diploids	Number of triploids
*C. gibelio*	G01	–	43 (11/4/14/14)
	G02	61 (16/21/15/9)	27 (7/8/5/7)
	G04	34 (10/9/6/9)	2 (0/1/0/1)
	G05	9 (1/2/5/1)	–
	G07	–	11 (8/2/0/1)
	G11	–	8 (4/3/1/0)

*C. auratus*	A01	5 (3/1/1/0)	–

*C. langsdorfii*	L01	1 (0/0/1/0)	–

*C. auratus *M line	M01	2 (0/1/0/1)	–

**Table 2:** Prevalence, abundance (mean ± standard deviation, SD), and intensity of infection (minimum–maximum) for parasite species in *Carassius gibelio*. P: prevalence, A: abundance, and I: intensity of infection.

	Parasite species	Diploid males			Diploid females			Triploid females		
		P (%)	A	I	P (%)	A	I	P (%)	A	I
			Mean (±SD)			Mean (±SD)			Mean (±SD)	
Monogenea	*Dactylogyrus dulkeiti*	100	55.29 (60.92)	6–353	100	59.48 (51.10)	6–291	100	48.68 (36.75)	12–187
	*Dactylogyrus intermedius*	100	45.02 (48.06)	8–301	100	43.88 (38.44)	3–214	100	39.30 (32.21)	5–196
	*Dactylogyrus anchoratus*	100	62.02 (69.52)	4–419	100	64.56 (60.44)	12–330	100	58.77 (53.23)	5–372
	*Dactylogyrus formosus*	100	17.29 (19.73)	1–118	98	19.02 (18.61)	0–93	100	16.68 (13.01)	1–90
	*Dactylogyrus inexpectatus*	100	17.88 (20.28)	2–125	100	15.81 (13.36)	2–76	100	13.56 (10.20)	1–73
	*Dactylogyrus vastator*	77	2.42 (3.45)	0–22	81	2.75 (2.65)	0–15	65	1.32 (1.81)	0–10
	*Dactylogyrus baueri*	46	7.98 (13.82)	0–66	56	5.23 (8.06)	0–46	63	5.89 (8.50)	0–39
	*Gyrodactylus sprostonae*	83	42.81 (135.46)	0–970	87	105.42 (267.80)	0–1387	87	18.85 (25.74)	0–180
	*Gyrodactylus shulmani*	44	2.79 (8.76)	0–62	42	2.56 (6.77)	0–35	23	0.26 (0.53)	0–3
	*Gyrodactylus longoacuminatus*	38	2.60 (9.02)	0–62	58	5.42 (13.99)	0–71	60	2.45 (3.63)	0–23
	*Paradiplozoon homoion*	8	0.12 (0.47)	0–3	13	0.17 (0.47)	0–2	7	0.08 (0.30)	0–2

Cestoda	Caryophyllidea sp.	2	0.02 (0.14)	0-1	4	0.73 (5.08)	0–37	–	–	–

Trematoda	*Aspidogaster* sp.	–	–	–	8	0.15 (0.63)	0–4	5	0.15 (0.73)	0–5
	*Diplostomum* sp.	–	–	–	–	–	–	1	0.01 (0.10)	0-1
	Digenea sp.	15	0.37 (1.24)	0–8	8	0.23 (0.87)	0–5	7	0.09 (0.35)	0–2

Nematoda	*Philometroides sanguinea*	23	0.92 (2.94)	0–20	25	2.40 (7.00)	0–46	45	7.98 (17.94)	0–103
	*Schulmanela petruschewskii*	17	3.46 (13.40)	0–72	31	10.27 (42.30)	0–299	22	4.89 (29.71)	0–280

Hirudinea	*Piscicola geometra*	2	0.02 (0.14)	0-1	2	0.02 (0.14)	0-1	4	0.04 (0.20)	0-1

Mollusca	Glochidium (*Unio *larval stages)	8	0.08 (0.27)	0-1	6	0.10 (0.45)	0–3	3	0.05 (0.31)	0–2

Protozoa	*Ichthyophthirius multifiliis*	58	11.52 (32.58)	0–162	65	6.56 (9.05)	0–36	44	9.09 (26.95)	0–192

Crustacea	*Ergasilus sieboldi*	15	0.31 (0.89)	0–5	21	0.62 (1.62)	0–8	13	0.32 (0.97)	0–6
	*Argulus foliaceus*	13	0.13 (0.34)	0-1	15	0.17 (0.43)	0–2	18	0.29 (0.94)	0–8

**Table 3:** The effects of ploidy, sex, year, and body size on parasite abundance (transformed in log). Parasite abundance was expressed by total parasite abundance, abundance of the most common parasitic groups, genera, or species in *Carassius gibelio*. The statistically significant *p* values are shown in bold.

Dependent variable	Predicted variables	*F*	*p*	Total *F*	Total *p*
Total parasites	Year	14.432	**<0.001**	10.038	**<0.001**
	Sex	0.002	0.960		
	Ploidy	0.002	0.964		
	Body size	11.572	**0.001**		
	Year *∗* ploidy	3.556	**0.015**		
	Ploidy *∗* body size	0.001	0.979		

Monogenea	Year	13.720	**<0.001**	9.119	**<0.001**
	Sex	0.010	0.922		
	Ploidy	0.206	0.651		
	Body size	6.660	**0.011**		
	Year *∗* ploidy	4.063	**0.008**		
	Ploidy *∗* body size	0.279	0.598		

*Dactylogyrus*	Year	8.387	**<0.001**	5.530	**<0.001**
	Sex	0.482	0.488		
	Ploidy	0.051	0.822		
	Body size	6.851	**0.010**		
	Year *∗* ploidy	1.927	0.127		
	Ploidy *∗* body size	0.069	0.794		

*Gyrodactylus*	Year	18.599	**<0.001**	9.557	**<0.001**
	Sex	0.110	0.740		
	Ploidy	0.038	0.846		
	Body size	1.446	0.231		
	Year *∗* ploidy	4.489	**0.005**		
	Ploidy *∗* body size	0.073	0.787		

*Ichthyophthirius multifiliis*	Year	11.887	**<0.001**	8.268	**<0.001**
	Sex	0.210	0.647		
	Ploidy	0.151	0.698		
	Body size	8.936	**0.003**		
	Year *∗* ploidy	1.847	0.140		
	Ploidy *∗* body size	0.191	0.662		

Nematoda	Year	12.201	**<0.001**	8.217	**<0.001**
	Sex	1.884	0.172		
	Ploidy	7.624	**0.006**		
	Body size	14.008	**<0.001**		
	Year *∗* ploidy	1.191	0.315		
	Ploidy *∗* body size	7.690	**0.006**		
